# Racial Differences in Left Ventricular Mass and Wave Reflection Intensity in Children

**DOI:** 10.3389/fped.2020.00132

**Published:** 2020-03-31

**Authors:** Kevin S. Heffernan, Wesley K. Lefferts, Nader H. Atallah-Yunes, Alaina C. Glasgow, Brooks. B. Gump

**Affiliations:** ^1^Department of Exercise Science, Syracuse University, Syracuse, NY, United States; ^2^Division of Academic Internal Medicine, Department of Medicine, University of Illinois-Chicago, Chicago, IL, United States; ^3^Division of Pediatric Cardiology, SUNY Upstate Medical University, Syracuse, NY, United States; ^4^Department of Public Health, Syracuse University, Syracuse, NY, United States

**Keywords:** children, vascular stiffness, wave intensity analysis, wave reflection, left ventricular mass

## Abstract

The burden of heart failure is disproportionately higher in African Americans, with a higher prevalence seen at an early age. Examination of racial differences in left ventricular mass (LVM) in childhood may offer insight into risk for cardiac target organ damage (cTOD) in adulthood. Central hemodynamic load, a harbinger of cTOD in adults, is higher in African Americans. The purpose of this study was to examine racial differences in central hemodynamic load and LVM in African American and non-Hispanic white (NHW) children. Two hundred sixty-nine children participated in this study (age, 10 ± 1 years; *n* = 149 female, *n* = 154 African American). Carotid pulse wave velocity (PWV), forward wave intensity (W1) and reflected wave intensity (negative area, NA) was assessed from simultaneously acquired distension and flow velocity waveforms using wave intensity analysis (WIA). Wave reflection magnitude was calculated as NA/W1. LVM was assessed using standard 2D echocardiography and indexed to height as LVM/[height (2.16) + 0.09]. A cutoff of 45 g/m (2.16) was used to define left ventricular hypertrophy (LVH). LVM was higher in African American vs. NHW children (39.2 ± 8.0 vs. 37.2 ± 6.7 g/m (2.16), adjusted for age, sex, carotid systolic pressure and socioeconomic status; *p* < 0.05). The proportion of LVH was higher in African American vs. NHW children (25 vs. 12 %, *p* < 0.05). African American and NHW children did not differ in carotid PWV (3.5 ± 4.9 vs. 3.3 ± 1.3 m/s; *p* > 0.05). NA/W1 was higher in African American vs. NHW children (8.5 ± 5.3 vs. 6.7 ± 2.9; *p* < 0.05). Adjusting for NA/W1 attenuated racial differences in LVM (38.8 ± 8.0 vs. 37.6 ± 7.0 g/m (2.16); *p* = 0.19). In conclusion, racial differences in central hemodynamic load and cTOD are present in childhood. African American children have greater wave intensity from reflected waves and higher LVMI compared to NHW children. WIA offers novel insight into early life origins of racial differences in central hemodynamic load and cTOD.

## Introduction

Although incidence and mortality from cardiovascular disease (CVD) is declining, there are still prominent disparities in CVD burden based on race (1). Compared to non-Hispanic whites (NHWs), African Americans have 33% higher death rates from CVD (1). Prevalence of hypertension in African Americans (~42–44%) is among the highest in the world and greater than that seen in NHWs (1, 2). As such hypertensive cardiac target organ damage (cTOD) is not only common but epidemic in African Americans (3, 4). African Americans have a 50% greater incidence of heart failure compared to their NHW counterparts (5).

cTOD occurs earlier in life in African Americans than in NHWs (6, 7) and is associated with premature CVD events (8). The CARDIA study reported that 26/27 deaths from heart failure occurred in young African Americans (<50 years of age) with only 1 NHW death (9). Racial differences in hypertension and hypertensive cTOD may have its origins in childhood as higher blood pressure (BP) in African American children track into young adulthood (10) and BP is a significant correlate of cTOD in African American children (11, 12). There are also racial differences in age-related increases in left ventricular mass (LVM) (13), a measure of cTOD, with African Americans having larger LVM in late childhood through young adulthood (14). Predictors of increases in LVM and development of LV hypertrophy from young adulthood to middle-age include larger LVM assessed in young adulthood, higher systolic BP and Black/African American race (15–17).

African Americans are more susceptible than NHW to BP-mediated cTOD suggesting that racial differences in hemodynamic load may have more profound effects on cTOD in African Americans (18, 19). Hemodynamic load is largely determined by central (large artery) stiffness and pressure from wave reflections (20, 21). Increases in arterial stiffness precede the development of hypertension in young adults (22) and increases in pressure from wave reflections have a profound and detrimental impact on the LV (20, 21). Increases in large artery stiffness and pressure from wave reflections alter ventricular-vascular coupling, contributing to increased afterload, myocardial strain, LVM, and ultimately LV hypertrophy (23). Racial differences in hemodynamic load may also have its origins in childhood as African American children have increased arterial stiffness compared to NHW children (24) and central BP is associated with LVM in young African American adolescents (25).

Wave intensity analysis (WIA) offers unique insight into ventricular-vascular coupling and central hemodynamic load. According to wave transmission-reflection theory, BP is an amalgam of traveling waves produced by the interaction of the LV with the aorta (input impedance/characteristic impedance) and the aorta with peripheral arteries (terminal impedance/vascular resistance). The propagation of traveling wavefronts in the systemic circulation encompass the exchange of the kinetic energy of blood flow and the potential energy of stored pressure in the elastic walls of the vessels. WIA is based on the relationship between the instantaneous change in pressure (dP) and the change in flow (dU) across the wave. When the LV contracts, this generates an initial forward pressure wave denoted as W1. WI represents a compression wave that accelerates both flow and pressure (26, 27). Once this wave reaches a bifurcation or area of impedance mismatch, the wave is partially reflected in the opposite direction from the distal circulation and travels back toward the heart. This backward traveling compression wave that decelerates flow but increases pressure is denoted by the negative area (NA) (26, 27). A second forward traveling wave is generated at the end of systole, mirroring the decompression (deceleration) of the wave produced by/contributing to the closing of the aortic value during diastole (26). This expansion wave, denoted as W2, is related to cardiac untwist and suction and thus LV relaxation kinetics. Measures obtained from WIA have been shown to correlate with LV structure and function (28, 29). WIA may thus offer a novel window into racial differences in cTOD risk in childhood.

The purpose of this study was to use WIA to assess racial differences in ventricular-vascular coupling and central hemodynamic load in children to gain insight into early life origins of cTOD. We hypothesized that African American children would have higher LVM, carotid artery stiffness and pressure from wave reflections.

### Methods

Two hundred sixty-nine children (154 African American, 149 female) from the Syracuse City community participated in this study as part of the Environmental Exposures and Child Health Outcomes (EECHO) study. Race was identified by parents/guardians as Black/African-American or non-Hispanic white (NHW). All participants were between 9 and 12 years of age, had a body mass >18 kg, and were free from any serious medical/developmental disabilities that would prevent them from participating in the study, as assessed by a health history questionnaire filled out by the guardian. Participants had no medical history of cancer, diagnosed hypertension, stroke, thyroid disease, pancreatitis, neuralgia, or diabetes. This study was approved by the institutional review board of Syracuse University and SUNY Upstate University. All parents/guardians and participants gave written consent and assent, respectively, prior to enrollment.

### Study Design

All cardiac and vascular measures were completed in a single visit and all visits occurred between 8:00 AM and 12:00 PM. Participants were asked to arrive in a fasted state. Cardiac measures were completed using standard 2-D echocardiography at SUNY Upstate Children's Hospital in the Division of Pediatric Cardiology. Vascular measures were conducted following a 5-min supine rest period in a dimly-lit, temperature-controlled laboratory (Human Performance Lab at Syracuse University) and consisted of brachial oscillometric pressures, carotid tonometric pressures, and simultaneous contralateral carotid Doppler ultrasonography.

Height and weight were assessed using a mechanical physician scale and stadiometer (Detecto, Webb City, MO). Body mass index (BMI) was calculated as weight (kg)/height (m)^2^. Socioeconomic status (SES) was calculated as an average of parental occupation, income, and education data (z-scored prior to averaging) as we have previously described (24, 30).

### Carotid Wave Intensity Analysis (WIA)

Images of the left common carotid artery (CCA) were obtained using Doppler ultrasound (ProSound α7, Aloka, Tokyo, Japan) and 7.5–10.0 mHz linear-array probe. Wave intensity analysis (WIA) combined with eTracking was used to derive measures of forward and reflected wave intensity and arterial stiffness. This method has been described in detail previously (31, 32). Briefly, this technique measures CCA distension waveforms (analogous to the pressure waveform) and flow velocity waveforms. The distance from the near-wall to far-wall lumen–intima interface was continuously traced using eTracking software. The echo-tracking system measures diameter changes within 1/16th of an ultrasound wavelength (0.013 mm) (28) creating a distension waveform almost identical to pressure waveforms (33). WIA distension waveforms were calibrated using carotid systolic and diastolic blood pressure. Pressure waveforms were obtained simultaneously in the contralateral CCA from a 10 s epoch using applanation tonometry (SphygmoCor, AtCor Medical, Sydney, Australia). CCA pressure waveforms were calibrated to simultaneously measured brachial mean arterial pressure (MAP) and diastolic BP (DBP) (measured using an oscillometric cuff) with MAP calculated as DBP + 0.33 × pulse pressure. Pulse pressure was calculated as systolic BP (SBP) minus DBP. End systolic pressure (ESP) was obtained from the carotid pressure waveform as the pressure at the incisura of the dicrotic notch. Flow velocity waveforms were measured using range-gated color Doppler signals averaged along the Doppler beam. An insonation angle ≤ 60° was maintained for all measures. Sample volume was adjusted to encompass the entire vessel. Wave intensity was calculated from an average of 5 beats using time derivatives of blood pressure (P) and velocity (U), where wave intensity = (dP/dt × dU/dt); thus, the area under the dP/dt × dU/dt curve represents the energy transfer of the wave (27).

WIA states that if these wavefronts carry a positive rate of pressure change, they are referred to as compression waves. Conversely, if the wavefront carries a negative rate of pressure change, they are referred to as expansion waves. It should be noted that “expansion” in this setting is an expression from fluid dynamics theory referring to “decreasing pressure” and not to be confused with “dilatation” (27). (1) W1 represents a forward compression wave produced during early systole that accelerates flow and increases pressure; (2) W2 represents a forward expansion wave that decelerates flow and reduces pressure; (3) the negative area (NA) between W1 and W2 is a backward traveling compression wave due to the sum of waves reflected from the periphery (wave reflection intensity) that decelerates flow but increases pressure. We additionally computed the reflection magnitude as NA/W1. Time from W1 to W2 (measured in milliseconds, ms) was taken as a proxy of ejection duration. A local one-point carotid pulse wave velocity (PWV) was calculated from WIA local wave speed as c = (dP/dU)/ρ where ρ is blood density and assumed constant (1,050 kg m^3^). Augmentation index (AIx) was calculated as the difference between the early and late systolic peaks of the distension waveforms to the total carotid pulse pressure and expressed as a percentage (P_max_-P_shoulder_/PP × 100). Reproducibility of carotid WIA has previously been demonstrated to be acceptable (32). Heart rate (HR) was obtained from simultaneous ECG from a single lead modified CM5 configuration.

### Echocardiography

Left ventricular mass (LVM) was derived from M-mode echocardiographic measurements of the septum, posterior wall and left ventricular diameter (Sonos 5500 Phillips, Andover, Massachusetts). Absolute LVM was calculated during diastole as: left ventricular mass = 0. 8 × |1.04[(left ventricular internal diameter + posterior wall thickness + septal thickness)^3^–(left ventricular internal diameter)^3^]) + 0.6 g|. This was based on consensus recommendations for chamber quantification (34). Relative wall thickness RWT was calculated as (2 × posterior wall thickness measured during diastole)/LV internal diastolic diameter. LV end-systolic wall stress (ESWS) was calculated as [0.334 × ESP × LV end-systolic diameter]/[LV posterior wall thickness at end systole × [1 + (LV posterior wall thickness at end systole/LV end-systolic diameter)] (35, 36); where ESP is carotid end-systolic pressure (37).

LVM was indexed (LVMI) using a variety of standard approaches [height, body surface area [BSA], and height raised to a power of 2.7]. There remains controversy regarding “best” methods to index LVM in children (and adults) as it is challenging to disentangle developmental growth from pathophysiological hypertrophy (38, 39). Aforementioned methods tend to “grossly” over diagnose left ventricular hypertrophy (LVH) in smaller individuals and underdiagnose LVH in larger overweight/obese individuals and this is particularly true for children <10 years of age and children of shorter stature (40, 41). Methods relying on BSA or a height index of 2.7 also do not factor in gender differences (42). Given that these strategies of indexing have been challenged for a variety of reasons (43), we chose to focus on LVM indexed using approaches put forth by Chirinos et al. and supported by Mehta in children (41, 42). Chirinos et al. and Mehta suggest indexing LVM to a height raised to a power of 1.7. This approach is a stronger predictor of CVD events in adults when compared to the traditional 2.7 method and may be more sensitive to obesity-mediated LVH in children (41, 42). We also employed a strategy put forth by Chinali et al. With the Chinali et al. approach, LVM is indexed to height raised to a power of 2.16 with a correction factor of + 0.09 and LVH defined using a single partition as values exceeding 45 g/m ^2.16^ (44). This approach has been suggested as more parsimonious strategy to index LVM in children as it does not require the need for time-consuming mathematical allometric scaling/ modeling or calculations of sex-specific, height-specific percentiles (44), offering a more clinically meaningful endpoint.

### Statistical Analyses

All data are reported as mean ± standard deviation with significance set a priori as *p* < 0.05. A chi-square test was used to test differences in sex distribution between groups. The effect of race on descriptive and cardiovascular measures (continuous variables) were assessed using analysis of variance (ANOVA). Analysis of covariance was used to test the effect of race on LVM after adjusting for potential confounding variables that emerged from ANOVA. Covariates included age, sex, SES, BMI percentile and carotid systolic pressure. We chose to adjust for carotid pressure rather than brachial pressure as central pressures more closely associate with cTOD (45). A second model additionally adjusted for heart rate. A third model adjusted for NA/W1 to explore the role of wave reflections as a potential mediator of racial differences in LVMI.

Mediational models were further tested using the SPSS PROCESS macro (v. 3.4) developed by Hayes (46). The indirect effects of race on LVMI through NA/W1 and HR was separately explored using the non-parametric bootstrapping procedure. These models were set to 1,000 bootstrap samples in order to minimize sampling error and for the estimation of bias corrected bootstrap confidence intervals for indirect effects. A 95% confidence intervals that does not contain 0 was used as criterion to establish significant mediation. All analyses were performed using Statistical Package for the Social Sciences (SPSS, version 26, Chicago, IL).

### Results

Descriptive characteristics are presented in Table 1. African American children were slightly older than NHW children (+ 0.3 yrs, *p* < 0.05) and had an overall lower SES-score (*p* < 0.05). African American and NHW children did not differ in height, BMI, BMI percentile, or gender (*p* > 0.05).

**Table 1:** Descriptive characteristics.

**Variable**	**African American *n* = 154**	**White *n* = 115**	***p*-value**
Age (years)	10.3 ± 0.9	10.6 ± 0.9	0.01
Female (%)	44	46	0.70
Height (cm)	145.4 ± 9.1	143.9 ± 8.4	0.18
Body mass index (kg/m^2^)	20.8 ± 5.7	20.1 ± 4.5	0.29
Body mass index (percentile)	69.0 ± 30.6	67.1 ± 30.4	0.62
Body surface area (m^2^)	1.32 ± 0.2	1.29 ± 0.2	0.31
Socioeconomic status (z-score)	−0.15 ± 0.7	0.42 ± 0.9	0.001

**Figure 1:**
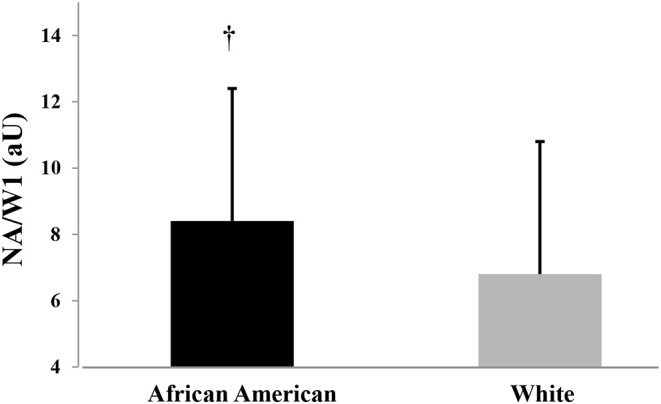
Wave reflection magnitude (NA/W1) in African American and White American Children; adjusted for age, sex, socioeconomic score, BMI percentile, and carotid systolic blood pressure. Significant group difference (*p* < 0.05).

Hemodynamic variables are displayed in Table 2. There was a trend for African American children to have higher brachial and carotid SBP compared to NHW children (*p* = 0.08 to 0.09). Groups did not differ in DBP (*p* > 0.05). African American children had higher carotid ESP compared to NHW children (Table 2, *p* < 0.05). African American and NHW children did not differ in carotid PWV, W1, W2 or AIx (*p* > 0.05). NA/W1 was higher in African American vs. NHW children (8.5 ± 5.3 vs. 6.7 ± 2.9; *p* = 0.001) and this was largely due to group differences in NA with African American children having higher NA compared to NHW (Table 2, *p* < 0.05). Group differences in NA/W1 remained after adjusting for age, sex, SES, BMI percentile, and carotid SBP (Figure 1, 8.4 ± 0.4 to 6.8 ± 0.4, *p* = 0.008). Adjusting for MAP instead of carotid SBP yielded similar overall findings (results not shown).

**Table 2:** Blood pressure and carotid wave intensity analysis (WIA).

**Variable**	**African American *n* = 154**	**White *n* = 115**	***p*-value**
Brachial systolic BP (mmHg)	115 ± 14	112 ± 10	0.08
Brachial diastolic BP (mmHg)	68 ± 6	67 ± 6	0.13
Mean arterial pressure (mmHg)	84 ± 8	82 ± 7	0.10
Carotid systolic BP (mmHg)	103 ± 10	101 ± 8	0.09
Carotid end-systolic pressure (mmHg)	85 ± 6	83 ± 7	0.03
Carotid diastolic BP (mmHg)	68 ± 6	67 ± 6	0.14
Carotid pulse wave velocity (m/s)	3.5 ± 4.9	3.3 ± 1.3	0.64
Carotid W1 (mmHg/m/s^3^)	9.2 ± 5.1	9.0 ± 4.1	0.72
Carotid W2 (mmHg/m/s^3^)	2.1 ± 1.2	2.2 ± 1.3	0.65
tW1-W2 (ms)	276 ± 73	262 ± 23	0.06
Carotid negative area (mmHg/m/s^2^)	71.3 ± 49.2	55.2 ± 27.9	0.002
Carotid augmentation index (%)	−3 ± 9	−4 ± 11	0.30
Heart rate (bpm)	73 ± 10	76 ± 10	0.03

**Figure 2:**
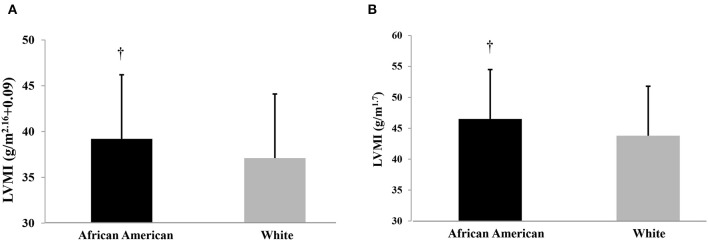
**(A)** Left ventricular mass index (LVMI) indexed to height of 2.16 in African American and non-Hispanic White Children; adjusted for age, sex, socioeconomic score, BMI percentile, and carotid systolic blood pressure. Significant group difference (*p* < 0.05). **(B)** Left ventricular mass index (LVMI) indexed to height of 1.7 in African American and non-Hispanic White Children; adjusted for age, sex, socioeconomic score, BMI percentile, and carotid systolic blood pressure. Significant group difference (*p* < 0.05).

Cardiac structural parameters are displayed in Table 3. According to chi-square analyses, the proportion of LVH was higher in African American vs. NHW children (25 vs. 12 %, *p* = 0.007). LVMI was higher in African American vs. NHW children when using height raised to a power of 2.16 + 0.09 (Figure 2A, 39.1 ± 8.0 vs. 37.2 ± 6.7 g/m^2.16^*, p* = 0.025) and when using height raised to a power of 1.7 (Figure 2B, 46.5 ± 9.8 vs. 43.9 ± 8.1 g/m^1.7^*, p* = 0.012); adjusted for age, sex, carotid systolic pressure, BMI percentile and socioeconomic status. Adjusting for MAP or carotid pulse pressure instead of carotid systolic pressure yielded similar overall findings (results not shown). Interestingly, additionally adjusting for either HR (38.9 ± 8.0 vs. 37.5 ± 7.0 g/m^2.16^; *p* = 0.17) or NA/W1 (38.8 ± 8.0 vs. 37.6 ± 7.0 g/m^2.16^; *p* = 0.19) attenuated racial differences in LVMI. Similar results were noted when indexing the LV to height. ^1.7^ Additionally adjusting for either HR (46.1 ± 10.0 vs. 44.3 ± 8.0 g/m^1.7^; *p* = 0.11) or NA/W1 (46.2 ± 9.8 vs. 44.2 ± 8.1 g/m^1.7^; *p* = 0.08) attenuated racial differences in LVMI. Mediational analyses with bootstrapping and PROCESS models revealed a significant indirect effect for HR (estimate = −0.76, 95% CI: −1.4, −0.25) and an indirect effect for NA/W1 that approached significance (estimate = −0.45, 95% CI: −1.0, 0.01) for LVMI^2.16^. Similarly, there was a significant indirect effect for HR (estimate = −0.63, 95% CI: −1.1, −0.2) and an indirect effect for NA/W1 that approached significance (estimate = −0.53, 95% CI: −1.2, 0.05) for LVMI^1.7^. RWT was slightly higher in the African American children (*p* = 0.07). This was driven by African American children having higher LV posterior wall thickness compared with NHW children (6.7 ± 1.0 vs. 6.4 ± 0.8 mm, *p* = 0.01). There were no racial differences in septal wall thickness (6.7 ± 1.0 vs. 6.7 ± 1.0 mm, *p* = 0.84). There were no racial differences in ESWS (Table 3, *p* > 0.05).

**Table 3:** Echocardiographic left ventricular properties.

**Variable**	**African American *n* = 154**	**White *n* = 115**	***p*-value**
Left ventricular mass (g)	88.2 ± 23.7	82.4 ± 18.8	0.031
Left ventricular mass/body surface area (g/m^2^)	65.9 ± 10.7	63.7 ± 10.2	0.08
Left ventricular mass/height (g/m)	60.1 ± 14.2	57.0 ± 11.1	0.052
Left ventricular mass/height^2.7^ (g/m^2.7^)	31.8 ± 6.4	30.7 ± 5.5	0.16
Left ventricular relative wall thickness	0.31 ± 0.05	0.29 ± 0.04	0.07
Left ventricular wall stress (kdynes/cm^2^)	49.7 ± 12.9	48.0 ± 12.8	0.30

### Discussion

This study set out to measure and compare LVM and carotid wave intensity in young African American and NHW children to gain insight into racial differences in early life origins of central hemodynamic load and cTOD. Our theoretical model was ground in an adult literature that strongly supports a physiological role for pressure from wave reflections as an effector of detrimental cardiac remodeling and LVH. Our findings add to this literature and reveal racial differences in LVMI and wave reflection intensity in children. Wave reflection intensity may partially contribute to racial differences in cTOD in children.

### Racial Differences in Left Ventricular Mass

LVM is an important measure of clinical prognosis and cTOD in childhood and offers insight into risk of CVD in early adulthood (43). LVM as a manifestation of cTOD is a powerful predictor of CVD risk in adults (47), particularly among African American adults (48). While the literature strongly supports racial differences in LVM in adults (with African Americans having higher LVM than NHW) (3, 4, 49), findings in children and adolescents are conflicting. When adjusting LVM to height, height^2.7^ or BSA, the majority of published studies note no racial differences in LVMI in children (11, 13, 50) although this is not a universal finding with select reports of greater LVM and wall thickness in African American children being noted (51, 52). Racial differences in LVM have been noted to occur longitudinally from late childhood through early adulthood (14) and may track into middle-age (16). When indexing LVM to height, height^2.7^ or BSA, we observed no racial differences in LVM in children. However, when utilizing alternative indexing strategies that have been proposed as more appropriate normalization methods (41, 42), we noted racial differences in LVMI (indexing height^1.7^ or height^2.16^) with African American children having larger LVMI that remained significant after statistical adjustment for age, sex, BMI percentile, SES, and central systolic BP. Future studies should consider using a variety of indexing methods when exploring the impact of race on cardiac dimensions and cTOD.

### Racial Differences in Central Hemodynamic Load

WIA may offer novel insight into early life origins of racial differences in central hemodynamic load and cTOD. We observed significantly greater wave reflection intensity in the carotid artery among African American compared to NHW children. There is a strong literature supporting a role for wave reflections in modulating LV load and subsequent LV remodeling. Animal models that employ use of large artery casting or surgical constriction to induce experimental increases in afterload consistently note increases in LVM and development of LVH (53, 54). Moreover, clinical studies in adults support cross sectional associations of wave reflections and subsequent pulse contour with LV geometry (55, 56). Alterations in supra-aortic (i.e., carotid) wave transmission and ensuing changes in carotid wave reflection intensity can alter LV loading sequence favoring increases in mid to late systolic load (57, 58). These wave reflection-based increases in cardiac load may precipitate cardiac remodeling over time as cardiac myosin heavy chain synthesis increases by nearly one third following exposure to a pressure overload (59). Moreover, hypertrophic cardiac remodeling appears particularly sensitive to wave reflections contributions to late rather than early systolic loading, independent of pressure (60). In our study, co-varying for NA/W1 attenuated racial differences in LVMI and NA/W1 approached significance as a statistical mediator. Our findings suggest that greater wave reflection intensity in African American children may serve as the substrate for increased afterload and thus partially contribute to racial differences in LVM.

Although African American children had higher wave reflection intensity and end-systolic pressure compared to NHW children (suggesting augmented late systolic load in African American children), there were no racial differences in LV end-systolic wall stress. Wall stress is considered an important stimulus for LV remodeling and a trigger of cardiac hypertrophy. It is possible that the slightly larger LVM in African American children is a compensatory adaptation aimed at defending LV systolic wall stress (35, 52). Over time and if left unchecked, detrimental LV remodeling may ensue contributing to noted racial differences in LVH and heart failure later in life.

While we noted racial differences in NA/W1 in children, there were no racial differences in carotid AIx. Methods that rely solely on the pulse contour as a means of appraising wave reflections are not without flaw. AIx offers some insight into global wave reflection magnitude in type A waveforms where there is a clear augmentation of pressure at the inflection point/ascending shoulder. However, as eloquently discussed by Mitchell (61), when AIx is > 0, augmented pressure from the pressure waveform alone represents just the “tip of the iceberg” because the majority of the reflected wave may be masked by the falling edge of the forward pressure wave (62, 63). In children with Type C waveforms (negligible/no augmentation of pressure above the shoulder), AIx may not capture wave reflection magnitude because calculated values are negative (P_2_ < P_1_). More than 75% of our sample had negative AIx values. AIx is also influenced by cardiac properties unrelated to wave reflections such as cardiac preload, LV stiffness, relaxation and suction as well as arterial reservoir function (64–67). Thus, methods that use both pressure and flow (like WIA to derive NA) are suggested as more optimal methodology to assess wave reflection magnitude (21, 68, 69) and our findings support use of WIA in children to assess cTOD risk.

There were no racial differences in carotid artery stiffness in children and this contrasts previous findings of racial differences in aortic stiffness in children (24). Although the aorta and carotid artery are generally both considered large elastic arteries, the ultrastructure of each is slightly different. Hemodynamic forces owing to hydrostatic effects may be different in the carotid artery vs. the descending-abdominal aorta (region of the aorta captured by cf-PWV) affecting stiffness (70). As such, each vessel is differentially modulated by CVD risk factors and aging over time. For example, the aorta stiffens more with aging and in response to obesity than the carotid artery, an effect exaggerated in the presence of high BP (71). It is possible that premature vascular aging may occur in the aorta prior to the carotid artery, with racial differences in carotid stiffness manifesting later in adolescence/young adulthood (72).

### Racial Differences in Left Ventricular Mass and Wave Reflection Intensity: Impact of Heart Rate

Reasons for racial differences in wave reflection intensity in children are unknown but may be related to underlying racial differences in HR. African American children had lower resting HR and this is a consistent finding reported in the literature. A meta-analysis of 17 studies found that African Americans have higher heart rate variability (HRV) compared to NHW, a pattern seen at all ages, suggesting greater cardiac vagal tone across the lifespan (73). This increased parasympathetic control of HR occurs concomitant with increased vascular sympathetic transduction and peripheral vascular resistance in African Americans (73). Interestingly, lower HR (and increased HRV) in African Americans has been seen to associate with larger LVM and development of LVH over time and has been suggested to magnify racial differences in LVM (16, 17, 51, 74, 75). In our study, covarying for HR attenuated racial differences in LVM and HR was a statistical mediator, supporting a role of HR as a partial effector of racial differences in LVM in children.

Lower HR in African American children may instigate greater wave reflection magnitude. There is an inverse association between HR and wave reflections. Although it assumed that this relationship only exists when examining global wave reflections with AIx (76), recent studies highlight HR dependency of wave reflection magnitude (assessed from wave separation analysis) as well (77, 78). A lower HR results in an increase in systolic ejection duration. Parenthetically, African American children had longer systolic ejection duration than NHW children. Without a change in vessel wall stiffness and wave speed, a longer ejection duration results in the confluence of the reflected wave and forward wave more likely occurring in late systole rather than early diastole. In the frequency domain, an increase in HR shifts the harmonics of the pulse waveform toward higher frequencies, an effect modulated by arterial viscoelasticity, resulting in an inverse frequency dependency of the reflection coefficient with HR (78). Thus, “paradoxical” findings reported in the literature of lower HR coexisting with increased LVM may be attributable to HR-mediated effects on wave reflections (79). Lower HR and longer ejection duration in African American children concomitant with increased wave reflection magnitude may increase late systolic load, contributing to ventricular-vascular uncoupling and LV remodeling.

### Limitations

Limitations to this study should be noted. This is a cross-sectional exploration of LVM and WIA in children. Thus, causation cannot be implied. It is possible that cardiac dysfunction may instigate altered wave propagation kinetics. For example, a larger LV may also be indicative of underlying LV dysfunction. Lower LV contractility may result in the genesis of a lower forward wave and by relation a lower absolute reflected wave (albeit possibly a higher relative reflected wave). However, we noted no racial differences in W1 and W2. This would support our theoretical model that higher wave reflection in African American children is likely not of cardiac origin (i.e., there were no differences in W1 to prompt racial differences in NA); wave reflection likely contributes to LVM via effects on LV load and loading sequence. 2D echocardiography may overestimate LVM in Black individuals due to bias in LV morphology assumptions use to calculate LVM. Future studies utilizing 3D echocardiography or cardiac MRI may be useful in further exploring racial variation in LVM in children and adolescents.

### Conclusion

In conclusion, African American children have greater wave reflection intensity and LVMI compared to NHW children of similar age and BMI. Premature ventricular-vascular uncoupling from increased wave reflection may instigate premature cTOD in African American children. Additional research is needed to explore the clinical implications of this finding, namely, whether targeting wave reflections in childhood may abrogate racial disparities in detrimental LV remodeling and heart failure later in life.

## Data Availability Statement

The datasets generated for this study are available on request to the corresponding author.

## Ethics Statement

This study was approved by the institutional review board of Syracuse University and SUNY Upstate University. Written informed consent to participate in this study was provided by the participants' legal guardian/next of kin.

## Author Contributions

KH and BG conceptualized study design, methodology, and performed statistical analyses. KH was the primary author and assisted with vascular data collection. WL collected all vascular data and assisted with data interpretation and manuscript preparation. NA-Y oversaw all cardiac echo measures and assisted with data interpretation and manuscript preparation. AG assisted with manuscript preparation. Funding for this study was through an NIH award to BG as PI.

### Conflict of Interest

The authors declare that the research was conducted in the absence of any commercial or financial relationships that could be construed as a potential conflict of interest.
